# Nanopore-Targeted Sequencing Improves the Diagnosis and Treatment of Patients with Serious Infections

**DOI:** 10.1128/mbio.03055-22

**Published:** 2023-01-18

**Authors:** Yi Zhang, Xuan Lu, Liang V. Tang, Linghui Xia, Yu Hu

**Affiliations:** a Institute of Hematology, Union Hospital, Tongji Medical College, Huazhong University of Science and Technology, Wuhan, China; College of Veterinary Medicine, Cornell University

**Keywords:** diagnosis, treatment, blood disease, nanopore-targeted sequencing, serious infection

## Abstract

Serious infections are characterized by rapid progression, poor prognosis, and difficulty in diagnosis. Recently, a new technique known as nanopore-targeted sequencing (NTS) was developed that facilitates the rapid and accurate detection of pathogenic microorganisms and is extremely suitable for patients with serious infections. The aim of our study was to evaluate the clinical application of NTS in the diagnosis and treatment of patients with serious infections. We developed an NTS technology that could detect microorganisms within a 6-h window based on the amplification of the 16S rRNA gene of bacteria, the internal transcribed spacer region of fungi, and the *rpoB* gene of Mycobacterium. The NTS detection results were compared with those of blood cultures and anal swabs from 50 patients with blood diseases suffering serious infections. The patient’s condition before and after NTS was compared. The response rate and the infection-related mortality after the adjustment of antibiotics based on NTS were calculated. The positivity rate of pathogens was highest in NTS (90%), followed by blood culture (32.6%) and anal swabs (14.6%). After adjusting antibiotics for bacteria and fungi detected by NTS, the patients’ condition improved significantly. Moreover, the response rate of anti-infective treatment based on NTS was 93.02% (40/43), and infection-related mortality was reduced to 0. NTS is an effective method to identify pathogens in the blood specimens of patients with serious infections and can guide anti-infection treatment and reduce infection-related mortality.

## INTRODUCTION

Severe infections have long puzzled clinicians, since they are characterized by rapid progression, poor prognosis, and difficulty in diagnosis. In particular, the number of patients with blood diseases who are susceptible to infections is increasing year by year. Due to technological progress, hematopoietic stem cell transplantation (HSCT) is now regarded as a standard treatment for a variety of blood disorders, including hematological malignancies, bone marrow failure syndromes, congenital immunodeficiencies, enzyme deficiencies, and hemoglobinopathies; however, it also has a significant risk of treatment-related mortality from conditioning regimen toxicity, graft versus host disease (GvHD), immunosuppression, and infections (1). Infections have a severe impact on patients with blood disorders, and acute infection compels clinicians to use wide-spectrum antibiotics before receiving blood culture results. However, administering anti-infective therapies relying on experience may lead to futile treatment, antimicrobial resistance, and even an increased risk of adverse outcomes and mortality. Thus, it is important to clearly identify the involved pathogens and use sensitive and effective antibiotics at an early stage to improve patient prognosis (2).

Current standard pathogen tests mainly include blood cultures, sputum cultures, and other tissue cultures. However, as some bacteria and fungi are difficult to grow in culture, the sensitivity of these inspection techniques is unsatisfactory (3) and not fit for purpose for acute serious infections, such as sepsis in patients with a blood disease, especially those undergoing HSCT. Moreover, the times required for Gram staining, organism identification (ID), and antimicrobial susceptibility testing for blood cultures are 1 day, 2 days, and 3 days, respectively (4, 5). The average turnaround time for anal swab samples, a common method for early detection of carbapenem-resistant Enterobacter (CRE) spp., is 2 to 4 days (6). Therefore, traditionally, it is difficult to obtain accurate evidence to adjust antibiotics in time, and a paradigm shift in microbiology diagnostics is urgently required to provide pathogen identification to clinicians before the onset of antibiotic treatment.

At present, some PCR-based diagnostic tests and high-throughput sequencing, called metagenomic next-generation sequencing (mNGS), are being applied alongside culture for the diagnosis of pneumonia and meningitis. However, mNGS involves shorter reads for metagenomic sequencing and is thus time-consuming and specimen dependent. Although mNGS can cover almost all pathogens present, its sensitivity is heavily affected by background gene level and it is susceptible to contamination by environmental species from the blood (7, 8), which prevents its application in clinical practice (9). Moreover, the overall turnover time of mNGS using the Illumina platform is about 24 h from sample preprocessing to nucleic acid extraction, library construction, sequencing, and analysis (10). Thus, PCR-based diagnostic tests and mNGS are not comprehensive enough to replace culture in clinical practice (11). Therefore, it seems pertinent and practical to set up systems for rapid microbiological detection to facilitate appropriate initial antibiotic treatment (12).

In this work, we developed a new targeted sequencing method called nanopore-targeted sequencing (NTS) based on the nanopore platform and targeting technology to address this diagnostic bottleneck. This method can detect pathogens based on the 16S rRNA gene of bacteria, internal transcribed spacer region (ITS) of fungi, and the *rpoB* gene of Mycobacterium spp. in a single test within a 20-h window for final reports and within 6 h for preliminary reports (13). The NTS technology is based on long read lengths (5,000 bp) and targeted amplification without interference from host background DNA (14). Thus, it has the features of speediness, economy, and accuracy and can therefore provide valuable clues for identifying pathogenic bacteria that cannot be detected by regular test methods. NTS has high sensitivity and specificity and can detect bacteria and fungi in a single trial, facilitating targeted therapy and decreasing the unnecessary and inappropriate use of antibiotics. In this article, we examine the response rate of NTS in clinical applications in preparation for its widespread use.

## RESULTS

### Patient characteristics.

A total of 53 patients were enrolled in this study, of which 3 were excluded because of insufficient information. The following describes the analysis of the remaining 50 patients. The basic clinical characteristics of the 50 patients are listed in Table 1. This study included 27 men and 23 women. Their mean age ± standard deviation was 35.04 ± 12.96 years. Primary diseases included acute lymphoblastic leukemia (32%), acute myeloid leukemia (24%), severe aplastic anemia (12%), myelodysplastic syndrome (8%), multiple myeloma (6%), lymphoma (6%), and others (12%). Most patients were sampled at the time of HSCT and agranulocytosis, and a large number received glucocorticoids (84%), immunosuppressive agents (78%), and arteriovenous catheterization (88%). The mean heart rate at the time of sampling was 109.14 ± 17.93 beats per minute, and the mean serum albumin was 32.87 ± 4.12 g/L. These indicators reflected the general condition of the patients at the time of sampling, which comprised fever, high inflammatory response, and slightly poor nutritional status. A total of five patients died in this study, four due to the recurrence of the primary disease and one because of primary graft failure.

**TABLE 1:** Patient characteristics

Characteristic	Category or value (mean ± SD or as indicated)^*a*^	No. of patients	% of patients
Gender	Male	27	54
	Female	23	46
Age (yrs)	35.04 ± 12.96		
Primary disease	ALL	16	32
	AML	12	24
	SAA	6	12
	MDS	4	8
	MM	3	6
	Lymphoma	3	6
	Other	6	12
HSCT	Yes	46	92
	No	4	8
Agranulocytosis	Yes	43	86
	No	7	14
Immunosuppressive agent	Yes	39	78
	No	11	22
Glucocorticoid	Yes	42	84
	No	8	16
Arteriovenous catheterization	Yes	44	88
	No	6	12
Heart rate (beats per minute)	109.14 ± 17.93		
Temp (°C)	>37.3	50	100
	≤37.3	0	0
Albumin (g/L)	32.87 ± 4.12		
No. or % of patients with indicated albumin value	≥35	17	34
	<35	33	66

a ALL, acute lymphoblastic leukemia; AML, acute myeloid leukemia; SAA, severe aplastic anemia; MDS, myelodysplastic syndrome; MM, multiple myeloma.

### Comparison of NTS, blood culture, and anal swab.

Table 2 presents the results of pathogen detection for each patient, using different methods at the same stage. The positivity rates of the three detection methods differed from each other. The positivity rate was highest for NTS (90%), followed by blood culture (32.6%) and anal swabs (14.6%). Multiple microorganisms can be detected simultaneously in a sample via NTS, with maximum reads of 62,120 and minimum reads of 21, whereas blood cultures and anal swabs can generally detect only one or two kinds of pathogenic microorganisms without quantitation. In addition, the anti-infection strategy was altered for most patients according to the NTS results, and the antibiotics administered and effectiveness of the adjustment are shown in Table 2.

**TABLE 2:** Pathogens detected by NTS, blood culture, and anal swabs

Patient	Pathogen(s) detected by NTS	No. of reads	Result from:		Antibiotic(s) based on NTS	Effective treatment adjustment
			Blood culture	Anal swab		
1	Candida albicans	2,399	Negative	Negative	Caspofungin	Yes
2	Lactobacillus iners	4,085	Negative	Negative	Meropenem	Yes
3	Acidovorax wautersii	159	Negative	Negative	Meropenem	Yes
4	Cryptococcus curvatus	2,865	Negative	Negative	Voriconazole	Yes
5	Kocuria varians	100	Negative	Negative	Meropenem, tigecycline	Yes
6	Negative	0	Negative	Negative		
7	Anaerococcus prevotii, Facklamia languida, Filobasidium uniguttulatum	213, 133, 1,484	Negative	Negative	Daptomycin	Yes
8	Staphylococcus haemolyticus	233	Negative	Negative	Linezolid	Yes
9	Abiotrophia defectiva, Kodamaea ohmeri	228, 2,574	Negative	Pseudomonas aeruginosa	Amphotericin B, caspofungin	No
10	Corynebacterium glaucum, Candida tropicalis	77, 1,336	Negative	Negative	Linezolid, caspofungin	Yes
11	Staphylococcus hominis, Corynebacterium ureicelerivorans	12,133, 630	Negative	Negative	Linezolid	Yes
12	Candida albicans	3,762	Negative	Negative	Micafungin	Yes
13	Pandoraea sputorum, Burkholderia gladioli	10,165, 4,740	No inspection	Negative	Cefoselis	Yes
14	Pseudomonas aeruginosa	335	Pseudomonas aeruginosa	No inspection	Piperacillin/tazobactam	Died
15	Negative	0	Escherichia coli	No inspection		
16	Negative	0	No inspection	Negative		
17	Paenibacillus glucanolyticus, Streptococcus pyogenes	426, 271	No inspection	Negative	Daptomycin	Yes
18	Negative	0	No inspection	Negative		
19	Lactobacillus iners, Candida parapsilosis	1,834, 1,376	No inspection	Pseudomonas aeruginosa	Ornidazole, amphotericin B	Yes
20	Corynebacterium pseudodiphtheriticum, Streptococcus mitis, Streptococcus pyogenes, Escherichia coli, Cutaneotrichosporon dermatis	1,426, 592, 110, 76, 13,088	Negative	Negative	Meropenem, tigecycline	Yes
21	Acinetobacter lwoffii, Cutaneotrichosporon curvatus	141, 4,414	Escherichia coli	Negative	Ceftazidime avibactam, voriconazole	Yes
22	Staphylococcus capitis, Enterobacter asburiae	754, 224	No inspection	Negative	Cefoperazone/sulbactam	Died
23	Staphylococcus saccharolyticus, Lactobacillus iners, Klebsiella variicola	530, 530, 156	Escherichia coli	Negative	Cefoperazone/sulbactam	Yes
24	Staphylococcus saccharolyticus, Staphylococcus epidermidis, Anaerococcus octavius, Escherichia coli	1,671, 1,311, 1,023, 65	Negative	Negative	Meropenem, teicoplanin	Yes
25	Acidovorax temperans, Prevotella nigrescens	609, 414	Negative	Negative	Cefoperazone/sulbactam	Yes
26	Finegoldia magna	292	Negative	Negative	Ornidazole, piperacillin/tazobactam	Yes
27	Corynebacterium pseudodiphtheriticum	710	No inspection	Negative	Meropenem	Yes
28	Corynebacterium tuberculostearicum, Lactobacillus iners, Pseudomonas putida	2,176, 510, 474	No inspection	Escherichia coli	Linezolid, minocycline	Yes
29	Alcaligenes faecalis, Escherichia coli, Meyerozyma guilliermondii, Yarrowia lipolytica	1,999, 867, 26,728, 14,115	Negative	Negative	Cefoperazone/sulbactam, caspofungin	Yes
30	Burkholderia gladioli, Pandoraea sputorum	7,325, 7,054	Negative	Negative	Meropenem, cefoperazone/sulbactam	Yes
31	Burkholderia gladioli, Anaerococcus octavius	474, 389	Negative	Negative	Cefoperazone/sulbactam	Yes
32	Kocuria salsicia	62,120	Escherichia coli	Negative	Linezolid	Yes
33	Neisseria mucosa, Burkholderia cepacia, Escherichia coli	722, 339, 97	Escherichia coli	Negative	Meropenem	Yes
34	Anaerococcus octavius, Corynebacterium aquatimens	615, 297	No inspection	Enterobacter cloacae, Klebsiella pneumoniae	Ornidazole	Yes
35	Paracoccus yeei, Streptococcus mitis, Corynebacterium jeikeium, Alternaria alternata	780, 305, 105, 2,320	No inspection	Negative	Imipenem	Yes
36	Staphylococcus saccharolyticus, Staphylococcus epidermidis, Burkholderia gladioli	3,525, 3,056, 510	No inspection	Negative	Daptomycin	No
37	Moraxella osloensis, Burkholderia gladioli	5,632, 307	Negative	Negative	Meropenem	Yes
38	Paracoccus speluncae	3,006	Negative	Escherichia coli	Imipenem	Yes
	Enterobacter cloacae	21		Enterobacter cloacae		
39	Corynebacterium ihumii	4660	Negative	Acinetobacter baumannii	Meropenem	Yes
40	Streptococcus agalactiae, Burkholderia multivorans, Escherichia coli	157, 12, 70	Negative	Negative	Meropenem	Yes
41	Bacillus cereus, Escherichia coli, Candida parapsilosis	8,180, 1,221, 3,256	Escherichia coli	Negative	Caspofungin, imipenem	Yes
42	Negative	0	Negative	Negative		
43	Micrococcus luteus, Pseudomonas aeruginosa, Alternaria alternata	158, 65, 12,122	Negative	Negative	Voriconazole, caspofungin, imipenem	Yes
44	Paracoccus sphaerophysae	602	Acidogens	Negative	Imipenem	Yes
45	Campylobacter showae, Treponema medium	106, 99	Negative	Negative	Tigecycline	Yes
46	Actinomyces odontolyticus, Aspergillus versicolor	1,425 3,709	Negative	Negative	Voriconazole, meropenem	Yes
47	Aspergillus versicolor	1,942	Negative	Enterobacter cloacae, Klebsiella pneumoniae	Amphotericin B	No
48	Escherichia coli, Rhodotorula glutinis	1,034, 12,478	Negative	Negative	Meropenem, voriconazole	Yes
49	Klebsiella pneumoniae	34	Negative	Negative	Meropenem	Yes
50	Burkholderia gladioli, Pandoraea sputorum	3,420, 73	Staphylococcus hominis	Negative	Piperacillin/tazobactam	Yes

Positivity rate (%)	90		23.1	14.6		
Treatment response rate						93.02 (40/43)^*a*^

a Value for effectiveness [% (no. successfully treated/no. whose treatment was adjusted)].

### Adjustment and effectiveness of antibiotics after NTS.

**FIG 1:**
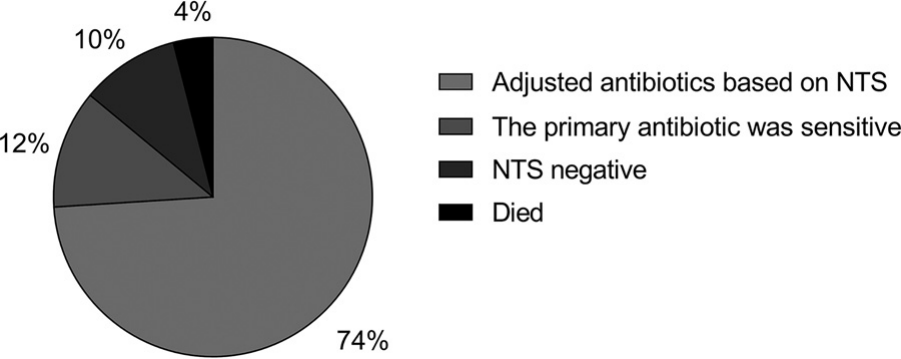
Adjustment of antibiotics after NTS. Antibiotics were adjusted according to the results of NTS in 74% of patients, while 26% of patients did not receive adjusted antibiotic regimens, among which 10% were due to negative results, 12% were because the previously administered antibiotics were effective against the newly detected pathogens, and 4% were because the patients died due to the recurrence of their primary diseases.

As shown by the results in Fig. 1, antibiotics were adjusted for 37 patients (74%) after NTS, whereas no adjustment was made for 6 patients (12%) because the bacteria detected were sensitive to the previously administered antibiotic and for 5 patients (10%) because of negative results. Two patients (4%) died because of the recurrence of the primary disease, so it was difficult to determine whether the antibiotic adjustment was effective in these cases. Overall, the response rate of anti-infection treatment was 93.02% (40/43) after the use of NTS in the HSCT center of Wuhan Union Hospital, significantly higher than that based on traditional etiological tests or empirical therapy during the same period. A 7-year single-center study reported that the cumulative response rate to antimicrobial therapy was 66.9% in HSCT patients, and the risk of dying from infection was 11.2% after allotransplantation and 0.8% after autotransplantation (15). At the same time, the infection-related mortality rate of patients in the HSCT center, which was 2.96% (9/304) in the previous 3 years in our center, decreased to 0 after the use of NTS. A comparison of the anti-infection response rate and infection-related mortality following treatment according to NTS versus traditional methods and experience is given in Table 3.

**TABLE 3:** Comparison of anti-infection response rate and infection-related mortality following the adjustment of antibiotics based on NTS versus traditional methods or experience

Parameter	Value (%)		
	When NTS was used	In another center^*a*^	In our center before NTS
Response rate	93.02	66.9	—^*b*^
Infection-related mortality	0	Allotransplantation, 11.2	2.96
		Autotransplantation, 0.8	

a Reference 15.

b —, The response rate in our center before NTS is missing.

### Comparison of patient status on the day of NTS and 7 days after the adjustment of antibiotics based on NTS.

**FIG 2:**
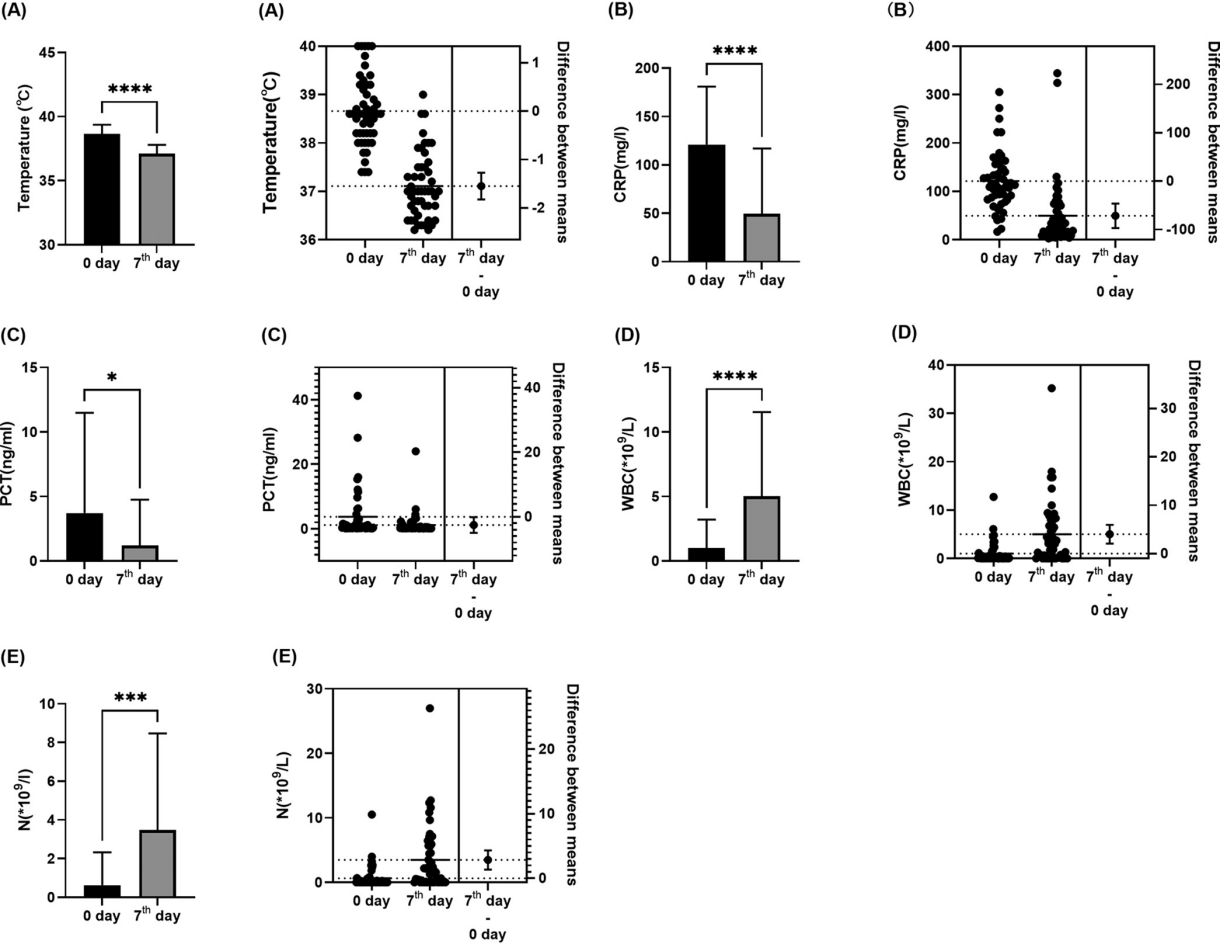
Comparison between patients on the day of NTS and on the 7th day after the adjustment of antibiotics based on NTS. (A) The temperature of patients was statistically significantly lower after NTS than before (*P* < 0.0001). (B) The CRP of patients was statistically significantly lower after NTS than before (*P* < 0.0001). (C) The PCT of patients was statistically significantly lower after NTS than before (*P* = 0.044). (D) The white blood cell count in patients was statistically significantly higher after NTS than before (*P* < 0.0001). (E) The neutrophil count was statistically significantly higher after NTS than before (*P* < 0.0002). Error bars show standard deviations. *, *P* < 0.05; ***, *P* < 0.001; ****, *P* < 0.0001.

The duration of remission was recorded based on the definition of effective anti-infective treatment in Materials and Methods. The average remission time of the patients undergoing NTS was found to be 7.00 ± 3.48 days. The body temperature, C-reactive protein (CRP), procalcitonin (PCT), white blood cell count, neutrophil granulocyte count, hemoglobin, and platelet count of patients on the day of NTS and the 7th day after the adjustment of antibiotics based on NTS were recorded, as shown in Table 4. The mean temperature at the time of sampling for NTS before the adjustment of antibiotics was 38.66 ± 0.71°C, and the mean values for CRP, PCT, white blood cell count, neutrophil granulocyte count, hemoglobin, and platelet count were, respectively, 121.10 ± 59.76 mg/L, 3.72 ± 7.77 ng/mL, (1.01 ± 2.19) × 10^9^/L, (0.63 ± 1.70) × 10^9^/L, 64.9 ± 20.01 g/L, and (41.74 ± 63.74) × 10^9^/L. On the other hand, the mean temperature on the 7th day after the adjustment of antibiotics according to NTS was 37.11 ± 0.68°C, and the mean values for CRP, PCT, white blood cell count, neutrophil granulocyte count, hemoglobin, and platelet count were, respectively, 49.47 ± 67.46 mg/L, 1.20 ± 3.56 ng/mL, (5.02 ± 6.52) × 10^9^/L, (3.48 ± 4.99) × 10^9^/L, 71.0 ± 14.63 g/L, and (59.39 ± 70.80) × 10^9^/L. After the adjustment of antibiotics based on NTS, the patients’ conditions improved significantly, especially regarding temperature, CRP, PCT, white blood cell count, and neutrophil granulocyte count, with statistically significat differences (*P* < 0.05) as shown in Fig. 2. Thus, after the use of NTS, antibiotic treatment was significantly improved compared to the previous period.

**TABLE 4:** Comparison of patients on the day of NTS and on the 7th day after the adjustment of antibiotics according to NTS

Parameter, time relative to adjustment of antibiotics^*a*^	Value (mean ± SD or high end/low end)	No. of patients	% of patients	*P* value
Temp (°C)				0.0001
Before	38.66 ± 0.71			
	>37.3	50	100	
	≤37.3	0	0	
After	37.11 ± 0.68			
	>37.3	15	30	
	≤37.3	35	70	
CRP (mg/L)				0.0001
Before	121.10 ± 59.76			
	>50	45	90	
	≤50	5	10	
After	49.47 ± 67.46			
	>50	17	34	
	≤50	33	66	
PCT (ng/mL)				0.044
Before	3.72 ± 7.77			
	>0.5	28	56	
	≤0.5	22	44	
After	1.20 ± 3.56			
	>0.5	15	30	
	≤0.5	35	70	
WBC (×10^9^/L)				0.0001
Before	1.01 ± 2.19			
	≥1	11	22	
	<1	39	78	
After	5.02 ± 6.52			
	≥1	32	64	
	<1	18	36	
Neutrophils (×10^9^/L)				0.0002
Before	0.63 ± 1.70			
	≥0.05	19	38	
	<0.05	31	62	
After	3.48 ± 4.99			
	≥0.05	36	72	
	<0.05	14	28	
Hb (g/L)				0.085
Before	64.9 ± 20.01			
	≥65	21	41	
	<65	29	58	
After	71.0 ± 14.63			
	≥65	32	64	
	<65	18	36	
PLT (×10^9^/L)				1.194
Before	41.74 ± 63.74			
	≥25	24	48	
	<25	26	52	
After	59.39 ± 70.80			
	≥25	34	68	
	<25	16	32	

a CRP, C-reactive protein; PCT, procalcitonin; WBC, white blood cell; Hb, hemoglobin; PLT, blood platelet.

### Typical cases in this study.

**FIG 3:**
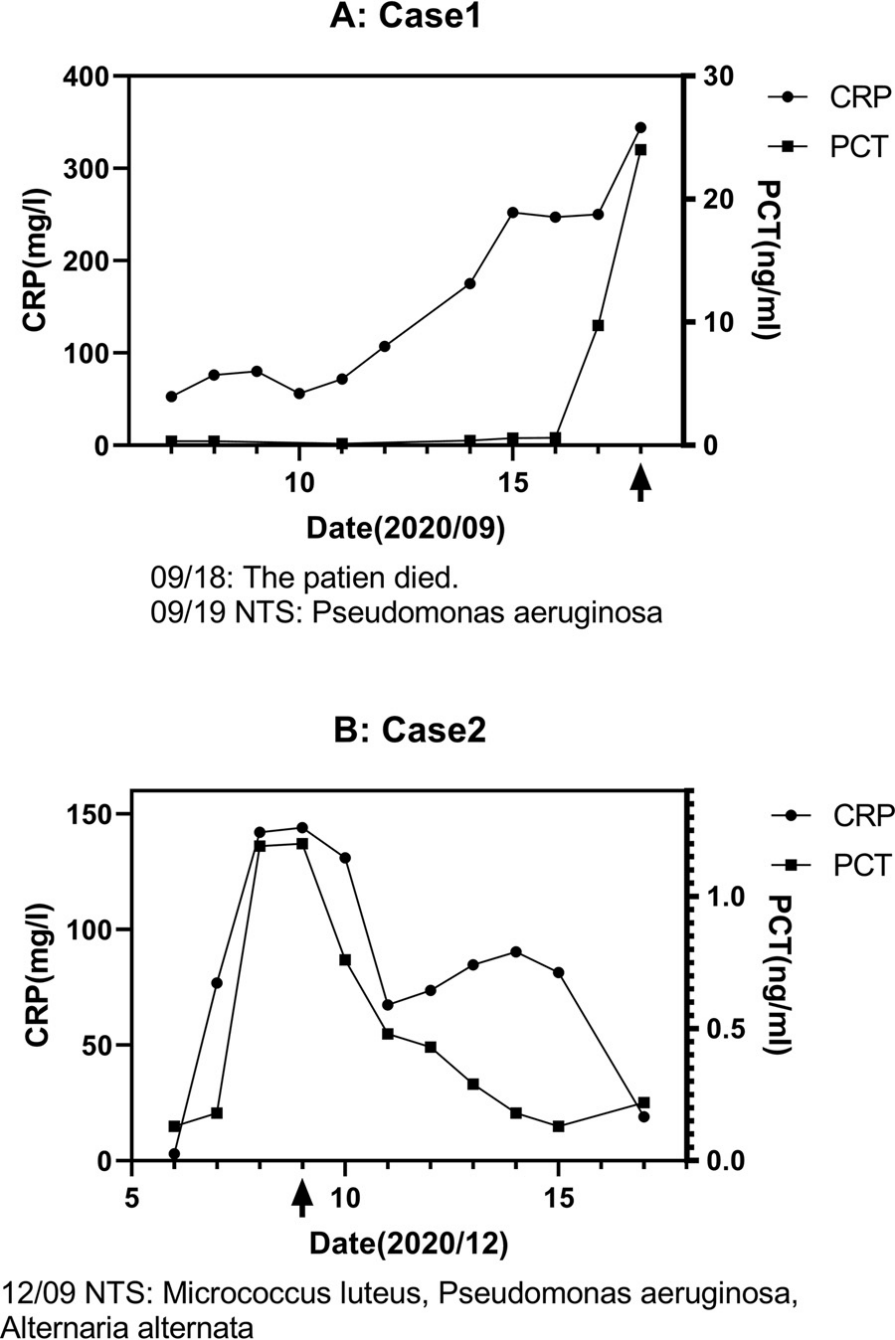
Typical cases in this study. Changes in the inflammatory indicators of typical patients under the guidance of NTS during hospitalization are shown.

Some of the patients in our study benefited significantly from NTS adjuvant etiology diagnosis and are listed herein. The changes in inflammatory biomarkers are shown in Fig. 3.

Case 1. A 25-year-old female patient whose primary disease was acute mixed-cell leukemia was admitted to the hospital on 7 September 2020. She had a severe infection after chemotherapy, and sputum culture on 12 September 2020 showed Acinetobacter lwoffii. However, the outcome was poor even though the antibiotics were adjusted, and inflammatory indicators continued to rise. At the same time, the patient’s blood culture returned negative results many times, so NTS testing was done on 18 September 2020. Nevertheless, the patient died due to a serious infection before the results came back on 19 September 2020. The NTS results suggested Pseudomonas aeruginosa infection. It is necessary to point out that the patient’s blood culture results from 20 September 2020 suggested that Pseudomonas aeruginosa was also present in the sample from 16 September 2020. However, by then, the patient was dead. If the NTS test had been performed as early as 16 September 2020 and the anti-infection treatment had been adjusted based on the results, the patient could have been saved.

Case 2. A 52-year-old male patient whose primary disease was Hodgkin lymphoma (HL) was admitted to the hospital on 30 November 2020. He underwent hematopoietic stem cell engraftment after conventional chemotherapy. Soon after, his infection worsened from 7 December 2020 onward, with a temperature of 38.2°C and CRP of 76.8 mg/L. Therefore, he was tested via NTS on 9 December 2020, with the results suggesting Micrococcus luteus, Pseudomonas aeruginosa, and Alternaria alternata infection. The antibiotics were then changed to voriconazole, linezolid, imipenem, and amphotericin B, and the patient was discharged successfully after improvement of the severe infection. Meanwhile, blood culture was performed on 8 December 2020, but the results on 12 December 2020 were negative. Therefore, if the patient had only been examined via blood culture, the antibiotic regimen could not have been adjusted effectively early on and he would not have recovered so smoothly.

## DISCUSSION

This study proposed a novel third-generation NTS technique for the diagnosis and treatment of infection in patients with serious infections. We described the etiological results for patients using NTS and found that NTS could detect a wide range of microorganisms in patients’ blood samples. Then, we compared the NTS results with those of blood cultures and anal swabs and discovered that NTS results based on samples from the same patient were more comprehensive and had a higher positivity rate, that is, the pathogen positivity rate was the highest in NTS (90%), followed by blood culture (32.6%) and anal swab (14.6%) samples. The positivity rate of blood cultures in our study was in accordance with a previous investigation showing that blood cultures before anti-infective treatment were positive for one or more microbial pathogens in 102 of 325 (31.4%) patients (16). At the same time, for all samples, the microbial species detected by NTS were more diverse. Therefore, NTS can detect certain bacteria or fungi that are difficult to culture or relatively rare in blood cultures and anal swabs. We evaluated the adjustment of antibiotics by clinicians based on the NTS results and found that the antibiotic regimens were effectively altered for most patients, showing improvements over the previous empirical anti-infection strategies. In summary, we pr posed the application of NTS, a novel and advanced method, for the clinical detection of pathogens in blood samples for the first time and evaluated the effectiveness of this detection method in assisting the diagnosis and treatment of infections in patients with serious infections. Thus, this study is innovative and provides a novel, effective solution for the issue of infections in patients with blood diseases, which progress rapidly and are dangerous and difficult to diagnose in clinical practice. Therefore, this study has commendable practicability and significance for clinical guidance.

The incidence of blood diseases is increasing year by year. HSCT has been widely used in the treatment of refractory and recurrent benign and malignant diseases of the blood, especially in patients with hematologic malignancies; however, there are several problems related to chemotherapy or the use of immunosuppressants and glucocorticoids after HSCT, such as bone marrow suppression and GvHD. Although HSCT centers provide a clean, hygienic, and protective environment, the risk of severe infection is still present (17) and may even lead to HSCT engraftment failure and death. It was reported that about 30% to 80% of patients developed a bacterial, fungal, or viral infection after receiving HSCT (18–20), and their cumulative incidences were 43.3%, 33.3%, and 23.3%, respectively (21). Bacterial infections always occur in the early graft period; both Gram-negative and Gram-positive bacteria may appear in this phase, while Haemophilus influenzae and Streptococcus pneumoniae are more frequent in the late period, as they are encapsulated pathogens (22). Bacterial bloodstream infections (BSIs), defined as the isolation of a bacterial pathogen from the blood obtained from peripheral blood or a central venous catheter (CVC), are an especially common complication in patients who received HSCT. Their incidence ranges from 18.6% to 43.6% depending on the study design, population, and transplantation protocol used (23). Moreover, BSIs are related to increased mortality after HSCT, and patients with BSIs tend to have longer stays in the hospital (16). A study demonstrated that very-early-phase BSI (infection occurring between 10 days before transplantation and 14 days after transplantation) was mainly associated with neutropenia, mucositis, and CVC, while late early BSI (infection occurring between 15 days and 100 days after engraftment) principally impacted the HSCT patients who were severely immunosuppressed with GvHD and corticosteroids (24). According to a literature review and investigation of the European Conference on Infections in Leukemia (ECIL), *Enterobacteriaceae* were isolated in 30% of HSCT patients with infection, coagulase-negative staphylococci in 24%, and enterococci in 8%; in the remaining cases, the pathogens included viridans streptococci (6%), Staphylococcus aureus (5%), Pseudomonas aeruginosa (5%), other Gram-positive pathogens (5%), and other Gram-negative pathogens (5%) (25). Our study found that at the beginning of infection in patients in the HSCT center, clinicians usually empirically used cefoperazone/sulbactam, fluconazole, and other antibacterial drugs that cover several bacteria and fungi. However, some patients still did not respond well to these anti-infective treatments, perhaps because they were infected with rare pathogens or those not covered by empirical antibiotic treatment. In Taiwan, most centers choose levofloxacin for the prophylaxis of Gram-negative bacilli, as recommended. Nevertheless, as HSCT patients receive chemotherapy, broad-spectrum antibiotics (which may change the microbiota), and immunosuppressive agents, the intestinal mucosa is severely damaged and may easily be infected by Clostridium difficile (5 to 30%) (26). At the same time, empirical antibiotic therapy could also increase the risk of bacterial drug resistance and secondary infection. An Italian prospective multicenter study has shown that prophylaxis using fluoroquinolones does not lower the infection risk of Gram-negative pathogens (27), and long-term antibiotic prophylaxis, especially with fluoroquinolone, could increase resistance in E. coli in HSCT patients (28, 29). Therefore, it is urgent and necessary to develop efficient, rapid, sensitive, specific, and economical etiological detection methods for clinical use.

However, at present, infection detection strategies in hematology patients mainly comprise blood culture, sputum culture, and other tissue cultures, which have limited sensitivity and detection range and high turnaround times. The combined use of such techniques with low sensitivity may lead to repeating sampling, increased treatment costs, and extended diagnosis time (30). Now, the enormous progress in sequencing-based diagnostic methods with broad detection ranges, such as mNGS technology, may contribute to addressing this problem (31). mNGS is characterized by deep sequencing and extensive bioinformatics to make clear host genome dominance and has the advantage of being unbiased and the disadvantages of low throughput, high costs, long turnaround times, and low sensitivity (the positivity rate for blood specimens was reported to be 88.98%) (32, 33). We planned a comparison between NTS and mNGS in the experimental plan, but the results were unsatisfactory because mNGS results were contaminated by background genes in the blood samples and the amounts of bacteria in many samples were difficult to detect in the process of running the machine. This reflects the fact that mNGS requires more pathogens, implying that its sensitivity is low. The targeted sequence detection technology detects sequences via amplified marker genes and requires less sequencing data and bioinformatics resources; thus, it can in theory reduce the cost and turnaround time. However, as with previous sequencing platforms with short read lengths, targeted sequencing technology still has limitations in discriminating pathogens because of the difficulty in obtaining complete sequence marker genes. Additionally, in this kind of platform, it is difficult to detect bacteria and fungi simultaneously because their different-sized marker genes are difficult to construct in the same sequencing library. Now, new technologies are emerging, mainly based on the nanopore sequencing platform, to improve the sensitivity and efficiency of detection. The function of such platforms is realized by an electrical signal conducted when DNA/RNA passes through a nanopore protein. Moreover, the nanopore protein does not select DNA/RNA sequences with specific lengths, and different lengths of DNA/RNA can pass through it completely. Thus, bacteria and fungi with different-sized marker genes can be included in the same sequencing library (34, 35). The NTS in our study, combining nanopore technology with targeted sequence detection, requires few sequencing data and bioinformatics resources and also has the advantages of real-time data output, end-to-end sequencing, and small-size nanopore sequencing, thus reducing costs and turnaround time. The turnaround time from the time the specimen is obtained from the patient to the time the report is derived is 6 to 20 h, where the preliminary 6 h includes 4 h for the experimental process and 2 h for sequencing and analysis. The experimental process includes nucleic acid extraction (1 h), multiplex targeted amplification (2 h), and library preparation (1 h). The sequencing and analysis time ranges from 2 h to 16 h depending on data size and computer performance. Therefore, in this technique, patients can get the primary detection results within 6 h and a full report within 20 h, greatly reducing the waiting time of patients for identifying pathogenic microorganisms and providing a guarantee for the immediate adjustment of antibacterial drugs (13). Additionally, since NTS technology is based on long read lengths and targeted amplification without host background DNA interference, its detection results have high accuracy. Therefore, NTS is more suitable for clinical applications and anti-infection treatment regimens based on NTS are more effective and comprehensive.

Although our research is sufficiently innovative, advanced, and practical, it has some deficiencies. First, this study originally planned to use first-generation sequencing methods, such as PCR and Sanger sequencing, as the gold standard to evaluate the sensitivity, specificity, predictive value, and other indicators of the application of NTS technology in the blood samples of patients with blood diseases complicated with serious infections. However, due to insufficient samples, it was not implemented, so only the positive detection results of NTS in these patients are described. In addition, we did not directly compare NTS and mNGS, for the reasons mentioned above. However, previous research has shown that the multiamplicon sequencing of NTS is more sensitive to low-copy-number severe acute respiratory syndrome coronavirus 2 (SARS-CoV-2) samples and requires minimal sequencing volume compared to the metagenomic and capture sequencing of mNGS (36). Second, because patients with bloodstream infections are more likely to die from bacterial and fungal infections and because cost-effective clinical assays for common viruses are available, our study did not include patients with viremia and we did not design targets for the detection of viruses. However, in subsequent versions of the NTS, we included viral targets for testing. Third, influenced by many factors, such as background nucleic acids and amplification efficiency, the “abundance of read” cannot be directly equated with the amounts of bacteria. Meanwhile, the pathogenicity of pathogens is related to the characteristics of the species itself, its abundance, and whether the pathogen survives or not. Therefore, the “abundance of read” is not directly related to the pathogenicity of pathogens. This is a general limitation of pathogenic testing methods based on the detection of nucleic acids of pathogens. Fourth, although NTS could report definite bacteria or fungi, which helped to provide effective anti-infection strategies, it was less effective than drug susceptibility tests in determining etiological resistance. At present, some recognized drug-resistance genes that can be used for detection have been proposed internationally, such as KPC genes and NDM genes (37). However, the effective combination of such data with NTS technology to guide clinical practice is worth further discussion. Besides this, studies on the improvement of patient prognosis and outcome after altering anti-infection strategies based on NTS should be further improved. We hope to develop a more rapid, efficient, sensitive, accurate, economical, and drug resistance-guided pathogen detection technology soon to better guide clinical practice and benefit patients. We also expect that our research will contribute to the development and improvement of this technology.

### Conclusion.

Our study introduces the application of NTS, a novel pathogen detection method, to blood samples of patients with serious infections. NTS can detect pathogens in blood specimens accurately and quickly, guiding clinicians to adjust anti-infection strategies and bringing significant benefits to patients. Although this study has some limitations, it also provides a basis for the emergence of faster, more sensitive, and more accurate pathogen detection technologies in the future.

## MATERIALS AND METHODS

### Research design and investigation.

A total of 53 patients with blood diseases who were hospitalized in the HSCT center and experienced severe infections between August 2020 and December 2020 at the Department of Hematology of Wuhan Union Hospital were included in this study. All patient data were obtained from NTS detection reports and the medical record database of the hospital. All patients provided informed consent for the use of their clinical data for research purposes, and this study was approved by the Ethics Committee of Wuhan Union Hospital.

**(i) Inclusion criteria.** All infected patients in the HSCT center were enrolled in this study with no restrictions on age, sex, primary disease, HSCT type, or regimen and regardless of whether antibiotics had been administered. The patients needed to meet one of the following conditions: *(a)* fever (body temperature >38°C) during the period of agranulocytosis (<0.5 G/L); *(b)* procalcitonin (PCT) ≥2.0 ng/mL, regardless of fever; or *(c)* CRP ≥50 mg/L, regardless of fever.

**(ii) Exclusion criteria.** Exclusion criteria included the following: *(a)* patients who refused to participate in blood collection; *(b)* fever caused by rabbit anti-human thymocyte immunoglobulin (ATG) or porcine anti-human lymphocyte immunoglobulin (ALG) during the conditioning regimen; or *(c)* fever due to implantation syndrome or acute GvHD (aGvHD) during granulocyte implantation or caused by other noninfectious factors.

**(iii) Research process.** The research process included the following:

*(a)* Recording the basic information of the patients, such as sex, age, primary disease, HSCT treatment, chemotherapy, glucocorticoids, immunosuppressants, arteriovenous catheterization, agranulocytosis, maximum body temperature before sampling, and status on the day of sampling, including white blood cell count, hemoglobin, platelet count, neutrophil count, CRP, and PCT.
*(b)* Collecting the NTS test findings, including the species and reads of the bacteria and fungi, and the corresponding results of the blood culture and anal swab samples.
*(c)* Recording adjustments in antimicrobial administration, blood temperature, white blood cell count, hemoglobin, platelet count, neutrophil count, CRP, and PCT after the NTS tests. Calculating the average number of days in which patients’ infection symptoms improved substantially after the NTS tests and antimicrobial adjustment. Comparing the changes in the above-described indicators of the patients before and after NTS tests and antibiotic adjustment.
*(d)* Calculating the treatment anti-infective response rate and infection-related mortality of patients after the adjustment of antibiotics according to the NTS tests.
*(e)* Describing the hospitalization of particular cases receiving NTS tests.

**(iv) Definition.** “Effective anti-infective treatment” was defined as follows: *(a)* The patient should have no fever (body temperature of <37.3°C) and *(b)* satisfy one of the two following conditions: *(1)* if both PCT and CRP were elevated before the inspection, PCT should be <0.5 ng/mL or CRP <20 mg/L, or *(2)* if only PCT or CRP was elevated before the examination, the parameter should be reduced to relatively normal values.

**(v) Instruments and equipment.** Laboratory assessments consisted of complete blood count, CRP, PCT, and anal swab samples. The blood cell count was assayed with the XN-9000 Sysmex hematology analyzer (Sysmex, Kobe, Japan) according to the manufacturer’s instructions. CRP was measured using an automated chemistry analyzer (SmartChem; Row2 Technologies, Inc., Parsippany, NJ, USA). PCT was analyzed using an automatic electrochemiluminescence immunoassay analyzer (Roche Cobas e411; Roche, Mannheim, Germany). Blood cultures were monitored using BacT/Alert bottles (bioMérieux, Marcy l’Etoile, France), and the growth of positive cultures was further characterized by matrix-assisted laser desorption ionization–time-of-flight mass spectrometry (Vitek MS; bioMérieux, France). Anal swab specimens were collected from patients and smeared on CHROMagar CRE screening medium. If CRE growth was detected, the strain was identified using an automated microbiological analysis system (Vitek 2 compact; bioMérieux, France), along with drug sensitivity tests, bacterial separation, and identification. The modified Hodge test was adopted in the carbapenemase phenotype test. All laboratory tests were assessed according to the Wuhan Union Hospital’s routine clinical laboratory procedures.

### NTS experimental methods.

**(i) Sample processing and DNA extraction.** Collected clinical samples were sent to the laboratory for DNA extraction within 4 h. The blood was first centrifuged at 50 × *g* for 10 min, and then the supernatant and top sediment were collected and centrifuged at 12,000 rpm for 10 min. Finally, 200 μL of sediment was used for DNA extraction. DNA extraction was performed using a Sansure DNA extraction kit (product number S1006; Sansure, Changsha, China).

**(ii) Amplification and nanopore-targeted sequencing.** Universal NTS primers were designed in-house to amplify the whole 16S rRNA gene of bacteria (1,500 bp), the whole ITS gene of fungi (400 to 800 bp), and part of the *rpoB* gene of Mycobacterium spp. (400 bp). The details of the primer design and PCR procedure are provided in reference 13. The amplification products of the 16S rRNA, ITS, and *rpoB* genes of the clinical samples and negative controls were pooled for sequencing library construction using the 1D ligation kit (product number SQK-LSK109; Oxford Nanopore Technologies). The sequencing was performed using the R9.4 flow cell on the GridION X5. The whole sequencing process lasted 2 to 16 h, and the sequencing data produced were analyzed by using a real-time bioinformatics analysis pipeline.

**(iii) Bioinformatics analysis pipeline and pathogen detection.** Base calling and quality control of sequencing raw data were done using the high-accuracy mode in Guppy (version 3.1.5). Reads with low quality (Q score of <7) and undesired length (<200 nucleotides [nt] or > 2,000 nt) were discarded. Clean reads were then mapped to 16S rDNA, ITS, and *rpoB* reference genes collected from the National Center for Biotechnology Information (NCBI) database. Up to 167 species of bacteria and 47 species of fungi can be mapped in this library. The E value of the basic local alignment search tool (BLAST) was set to 1e−5, and the taxonomy of each read was assigned according to the taxonomic information of the mapped subject reference with the highest identity and coverage. A consensus sequence of the reads assigned to the same species was generated by using Medaka (version 0.10.1), and the consensus was remapped to the reference database. The species-level taxonomy of the mapped subject reference was detected as the final result.

The pathogen detection results of the clinical samples were interpreted according to a strict set of rules that are described in detail in reference 13.

### Statistical methods.

All statistical analyses were conducted with the IBM SPSS Statistics 22 statistical software. Continuous variable data having a normal distribution are represented by mean values and standard deviations, and intergroup comparison was performed with the independent-sample *t* test. Descriptive statistics are recorded in percentages (%), and the statistical results of the descriptive studies were compared directly.
