# Correction for Zhang et al., “Nanopore-Targeted Sequencing Improves the Diagnosis and Treatment of Patients with Serious Infections”

**DOI:** 10.1128/mbio.02461-23

**Published:** 2023-10-24

**Authors:** Yi Zhang, Xuan Lu, Liang V. Tang, Linghui Xia, Yu Hu

## AUTHOR CORRECTION

Volume 14, no. 1, e03055-22, 2023, https://doi.org/10.1128/mbio.03055-22. Page 11, last two lines: “this study was approved by the Ethics Committee of Wuhan Union Hospital.” should read “this study was approved by the Ethics Committee of Wuhan Union Hospital under ethics approval number [2023] No.0170.”

Page 13, the following statement should be added to Acknowledgments: “We gratefully acknowledge the technical support provided by the team of Tiangang Liu at Wuhan University and Wuhan Dgensee Clinical Laboratory Co., Ltd.”

